# Evaluation of cellular uptake and intracellular trafficking as determining factors of gene expression for amino acid-substituted gemini surfactant-based DNA nanoparticles

**DOI:** 10.1186/1477-3155-10-7

**Published:** 2012-02-01

**Authors:** Jagbir Singh, Deborah Michel, Jackson M Chitanda, Ronald E Verrall, Ildiko Badea

**Affiliations:** 1 Drug Design and Discovery Research Group, College of Pharmacy and Nutrition, University of Saskatchewan, 110 Science Place, Saskatoon, S7N 5C9, Canada; 2 Department of Chemistry, University of Saskatchewan, 110 Science Place, Saskatoon, S7N 5C9, Canada

**Keywords:** cellular uptake, endosomal escape, non-viral gene delivery, clathrin-mediated endocytosis, caveolae-mediated endocytosis

## Abstract

**Background:**

Gene transfer using non-viral vectors offers a non-immunogenic and safe method of gene delivery. Cellular uptake and intracellular trafficking of the nanoparticles can impact on the transfection efficiency of these vectors. Therefore, understanding the physicochemical properties that may influence the cellular uptake and the intracellular trafficking can aid the design of more efficient non-viral gene delivery systems. Recently, we developed novel amino acid-substituted gemini surfactants that showed higher transfection efficiency than their parent compound. In this study, we evaluated the mechanism of cellular uptake of the plasmid/gemini surfactant/helper lipid nanoparticles and their effect on the transfection efficiency.

**Results:**

Nanoparticles were incubated with Sf 1 Ep cells in the presence of different endocytic inhibitors and gene expression (interferon-γ) was measured using ELISA. Clathrin-mediated and caveolae-mediated uptake were found to be equally contributing to cellular internalization of both P/12-7NH-12/L (parent gemini surfactant) and P/12-7NGK-12/L (amino acid-substituted gemini surfactant) nanoparticles. The plasmid and the helper lipid were fluorescently tagged to track the nanoparticles inside the cells, using confocal laser scanning microscopy. Transmission electron microscopy images showed that the P/12-7NGK-12/L particles were cylindrical while the P/12-7NH-12/L particles were spherical which may influence the cellular uptake behaviour of these particles. Dye exclusion assay and pH-titration of the nanoparticles suggested that high buffering capacity, pH-dependent increase in particle size and balanced DNA binding properties may be contributing to a more efficient endosomal escape of P/12-7NGK-12/L compared to the P/12-7NH-12/L nanoparticles, leading to higher gene expression.

**Conclusion:**

Amino-acid substitution in the spacer of gemini surfactant did not alter the cellular uptake pathway, showing similar pattern to the unsubstituted parent gemini surfactant. Glycyl-lysine substitution in the gemini spacer improved buffering capacity and imparted a pH-dependent increase of particle size. This property conferred to the P/12-7NGK-12/L nanoparticles the ability to escape efficiently from clathrin-mediated endosomes. Balanced binding properties (protection and release) of the 12-7NGK-12 in the presence of polyanions could contribute to the facile release of the nanoparticles internalized via caveolae-mediated uptake. A more efficient endosomal escape of the P/12-7NGK-12/L nanoparticles lead to higher gene expression compared to the parent gemini surfactant.

## Background

Gene therapy is based on the delivery of therapeutic genes to prevent or treat a disease. The method includes replacing a nonfunctional gene, introducing a new or missing gene, silencing a gene, or regulating gene expression. Gene-based therapy could offer an improved therapeutic solution and a cost-effective option to the treatment of many diseases, including cancer and infectious diseases [1,2]. Among the available gene transfer technologies, non-viral vectors offer a non-immunogenic and safe method of gene delivery. However, they have generally lower transfection efficiency compared to their viral counterparts.

**Figure 1:**
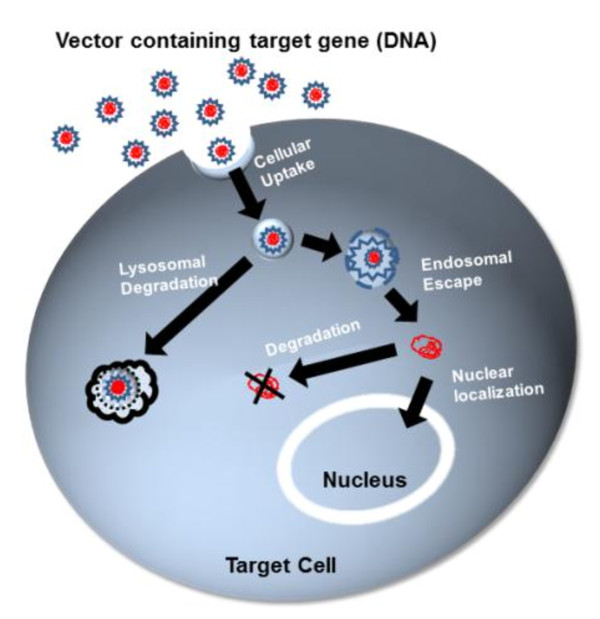
**Intracellular trafficking of DNA-delivery vector complexes**. This schematic representation indicates the critical barriers in successful gene delivery: cellular uptake, endosomal escape and nuclear localization. A delivery vector interacts with the cell membrane for internalization. Once inside the cell, the delivery vector should facilitate endosomal escape and avoid DNA degradation in lysosomes. Cytoplasmic stability of plasmid DNA and localization in the nucleus are final steps to successful gene transfer.

For successful gene expression, a delivery vector needs to overcome three major challenges (Figure 1): cellular uptake, endosomal/lysosomal escape and nuclear localization [3]. Cellular uptake is an important process, as it determines the number of particles that are internalized and available for gene expression. Moreover, the mechanism of uptake may determine the intracellular pathway and the final fate of the vectors [4]. Clathrin-mediated, caveolae-mediated uptake and macropinocytosis are the most common uptake pathways utilized by mammalian cells to engulf macromolecules or solutes impermeable to plasma membrane [4]. We assessed the effect of these three cellular uptake pathways on the gene transfer efficiency of the gemini surfactant-based nanoparticles. The clathrin-mediated uptake involves special membrane structures called clathrin-coated pits [5]. When ligands bind to these receptors, the coated pits form a polygonal clathrin lattice with the help of adaptor proteins. These clathrin-coated pits are pinched off from the plasma membrane and internalized to form intracellular clathrin-coated vesicles ranging in size from 100 to 150 nm in diameter [5]. Inside the cell, the clathrin coat depolymerizes to form early endosomes which then fuse with late endosomes and proceed to finally fuse with lysosomes. Particles internalized via this pathway experience a drop in pH, towards acidic conditions (pH 5-6), as they travel towards late endosomes, before merging with lysosomes [6]. Chlorpromazine and potassium depletion can dissociate clathrin from the surface membrane and inhibit clathrin-mediated endocytosis [7,8]. Caveolae-mediated uptake is another important pathway that involves small hydrophobic domains that are rich in cholesterol and glycosphingolipids [9]. Contrary to clathrin-mediated uptake, the caveolae-dependent pathway follows a non-acidic and non-digestive intracellular route. Filipin III inhibits caveolae-mediated uptake by binding to 3β-hydroxysterol, a major component of glycolipid microdomains and caveolae [10]. Genistein also inhibits caveolae-mediated uptake by local disruption of the actin network and by preventing the recruitment of dynamin II, both necessary for this type of cellular uptake [11]. Water-soluble methyl-β-cyclodextrin forms inclusion complexes with cholesterol and is known to inhibit both clathrin-mediated and caveolae-dependent uptake by depleting cholesterol from the plasma membrane [12-14]. Macropinocytosis is a non-selective internalization of large volumes of extracellular medium through cell membrane protrusions that collapse onto and fuse with the cell membrane [15]. The large endocytic vesicles are neither coated nor concentrated before internalization. Phosphatidylinositol 3 kinase and rho family GTPase activities influence macropinocytosis by regulating actin rearrangements. Wortmannin, a phosphatidylinositol 3 kinase inhibitor, can be employed to inhibit macropinocytosis [16].

Understanding the physicochemical properties of the gene delivery vectors in association with these uptake pathways can lead to rational design of nanoparticles that target those pathways and improve the intracellular fate of the particles. For example, the design of delivery agents that respond to the change in pH to escape from endosomes so as to evade degradation in lysosomes can improve the transfection efficiency of these non-viral vectors [17]. To capitalize on the pH sensitivity of these nanoparticles, clathrin-mediated uptake should be targeted to utilize this phenomenon.

**Figure 2:**
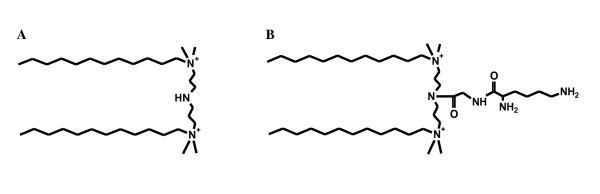
**Chemical structure of gemini surfactants**. (A) Parent unsubstituted gemini surfactant (12-7NH-12) and (B) glycyl-lysine substituted gemini surfactant (12-7NGK-12).

Our research group has focused on the optimization of cationic N,N-bis(dimethylalkyl)-α,ω-alkanediammonium gemini surfactants or m-s-m; where m and s refer to the number of carbon atoms in the alkyl tails and in the polymethylene spacer groups, respectively [18,19]. More recently, a series of third generation gemini surfactants have been developed by introducing an amino acid/dipeptide group at the N position of the spacer of a previously studied compound (12-7NH-12) (Figure 2A). The glycyl-lysine substituted derivative (12-7NGK-12, Figure 2B) achieved the highest gene expression among the series, performing significantly better than the unsubstituted parent compound [20,21].

In this study, we evaluated the cellular uptake behavior of plasmid/gemini/lipid (P/G/L) nanoparticles and investigated their impact on the transfection efficiency of these nanoparticles. We also explored the physicochemical properties that may have led to the difference in cellular uptake and intracellular trafficking of these nanoparticles resulting in the difference in transfection efficiency between these vectors.

## Materials and methods

### Reagents for uptake studies

Genistein, filipin III, methyl-β-cyclodextrin, chlorpromazine hydrochloride and wortmannin were purchased from Sigma-Aldrich, Oakville, Canada.

### Formulations

A model plasmid pGT.IFN-GFP that encodes for interferon (IFN)-γ and green fluorescent protein was used for all formulations. Gemini surfactants were formulated with pGT.IFN-GFP in the presence of a helper lipid 1,2-dioleoyl-sn-glycero-3-phosphoethanolamine (DOPE; Avanti Polar Lipids, AL, U.S.A.) creating P/G/L nanoparticles. A plasmid to gemini surfactant charge ratio of 1:10 was used for all formulations (as described earlier [20]). A quantity of 100 ng plasmid DNA per well was used in the 96-well plates for transfection.

### Cell culture and propagation

Cotton tail rabbit epithelial cells, Sf 1 Ep (CCL-68, ATCC, VA, USA) were grown in Minimum Essential Medium (MEM, GIBCO, NY, USA) supplemented with 10% fetal bovine serum and antibiotic/antimycotic agents, respectively. All cells were grown to 70-80% confluency. The cells were seeded at a concentration of 20,000 cells per well in 96-well tissue culture plates (Falcon BD, Mississauga, Canada) and were incubated for 24 hours at 37°C/5% CO_2 _prior to transfection.

### Cell toxicity assay

Genistein (200 μM), filipin III (5 μg/ml and 1 μg/ml), methyl-β-cyclodextrin (10 mM and 5 mM), chlorpromazine hydrochloride (10 μg/ml and 5 μg/ml), wortmannin 50 nM were incubated with the cells for 1, 2, 3 and 4 hours [12,22-24]. For potassium depletion, the cells were washed three times with potassium free buffer containing 140 mM NaCl, 20 mM Hepes pH 7.4, 1 mM CaCl_2_, 1 mM MgCl_2 _and 1 mg/ml D-glucose, followed by 2 minutes incubation with hypotonic buffer (potassium free buffer diluted with water 1:1) [8,22] for 1, 2, 3 and 4 hours. At the end of the incubation, cells were washed three times with PBS and incubated with fresh supplemented media at 37°C/5% CO_2 _for 24 hours. A 10 μl aliquot of 5 mg/ml (3-(4,5-dimethylthiazol-2-yl)-2,5-diphenyltetrazolium bromide (MTT, Invitrogen, Burlington, Canada) aqueous solution was added in each well and the plates were incubated at 37°C/5% CO_2 _for 3 hours. Supernatants were removed and the cells were washed with PBS. The formed purple formazan was dissolved in 200 μL dimethyl sulfoxide (DMSO, Sigma) on a plate shaker for 10 minutes. The plates were incubated for 10 minutes at 37°C to eliminate air bubbles. Absorbance was measured at 550 nm using a plate reader (Biotek Microplate Synergy HT, VT, USA). Cell toxicity of the chemically treated/potassium depleted cells were expressed as a percentage of dead cells compared to the untreated normal cells.

$$\%\;dead\;cells=\frac{Abs_{control}-Abs_{treated}}{Abs_{control}}100$$

### Transfection

In a 96-well plate, cells were incubated (in triplicate) for 30 minutes with genistein 200 μM, methyl-β-cyclodextrin 5 mM, wortmannin 50 nM, filipin III 5 μg/ml and for 60 minutes with filipin III 1 μg/ml, chlorpromazine hydrochloride 5 μg/ml and potassium free buffer prior to addition of P/G/L nanoparticles. The nanoparticle formulations were prepared as described above and incubated with the cells for 2 hours. After 2 hours, the media was removed and cells were washed three times with PBS and replenished with fresh media. Supernatants were collected at 24, 48 and 72-hour intervals. At 72 hours, an MTT assay was performed to determine the combined toxicity of P/G/L nanoparticles and chemical treatment/potassium depletion on cell viability. ELISA was used to detect the IFN-γ secreted into the supernatant and was performed according to the BD Pharmingen protocol. Protein concentration was calculated from a standard curve using recombinant IFN-γ.

### Confocal laser scanning microscopy (CLSM)

The model plasmid pGT.IFN-GFP was labeled with tetramethyl-rhodamine (ex/em wavelengths: 546 nm/576 nm) using Label IT^® ^Tracker™ Kit (Mirus, WI, U.S.A.) according to the manufacturer's protocol. N-(7-nitro-2-1,3-benzoxadiazol-4-yl) labeled DOPE (ex/em wavelengths: 460 nm/535 nm) was purchased from Avanti Polar Lipids, AL, U.S.A.

P/G/L nanoparticles were prepared as described in transfections using tetramethyl-rhodamine labeled plasmid with 10% of DOPE in the formulation being substituted with fluorescent tagged DOPE.

Sf 1 Ep cells were treated with chemical inhibitors - genistein, chlorpromazine, wortmannin and methyl-β-cyclodextrin as described under the section 'Transfection', prior to incubation with fluorescent P/G/L nanoparticles. Additionally, an aliquot of 30 μM DAPI was added to the cells for staining the nucleus. After 2 hours, the cells were washed with PBS three times and images were taken using a Leica TCS SP5 laser scanning confocal microscope (Leica Microsystems Inc., Benshein, Germany). LAS AF Lite 2.4.1 (Leica Microsystems CMS GmbH) and ImageJ 1.44 p (National Institute of Health, U.S.A.) were used for image processing.

### pH titration and particle size measurement

P/12-7NH-12/L and P/12-7NGK-12/L nanoparticles were prepared as described previously. An MPT-2 autotitrator connected to a ZetaSizer Nano ZS (Malvern Instruments, Worcestershire, UK) was used to determine changes in particle size as a function of pH. Briefly, P/G/L nanoparticles (9 ml) were placed in the titration cell of the auto-titrator and titrated over basic (0.05 M NaOH) to acidic (0.1 M HCl) pH range at 0.2-0.5 pH unit intervals.

### DNA/gemini surfactant interactions

Nanoparticles were prepared using plasmid and gemini surfactants at a +/- charge ratio of 10 in the presence of DOPE as described earlier, with samples prepared in quadruplicate. P/G/L nanoparticles were incubated with ethidium bromide (1 μg/mL) and heparin buffer (20 mM HEPES, 5.2% glucose, 0 U/ml, 5 U/ml, 10 U/ml or 20 U/ml heparin, pH 7.0) for 10 minutes at room temperature. Fluorescence excitation was carried out at 528/20 nm and emission measured at 590/35 nm using a multiplate reader (Biotek Microplate Synergy HT, VT, U.S.A.). The relative fluorescence of the P/G/L complexes was expressed as a percentage of the fluorescence of a plasmid solution in presence of corresponding (0 U/ml, 5 U/ml, 10 U/ml, 20 U/ml) heparin buffer.

$$\%\;fluorescence=\frac{\left(fluorescence_{P/G/L\;with\;heparin}-fluorescence_{P/G/L}\right)}{fluorescence_{plasmid\;in\;presence\;of\;buffer}}100$$

### Transmission electron microscopy (TEM)

Formulations were prepared as described earlier and loaded onto formvar-coated 300-mesh copper grids using 5 μL aliquot of each sample. The samples were incubated for 2 minutes and the liquid was blotted with absorbent tissue. The specimens were stained with 1% phosphotungstic acid for 15 seconds, and blotted with tissue. The dried samples were examined with a Philips CM 10 electron microscope at an accelerating voltage of 100 kV, and pictures taken on 31/4″ × 4″ Kodak Electron Microscope Film 4489.

### Statistical analyses

Results are expressed as the mean of n ≥ 3 ± standard deviation. One way analysis of variance (ANOVA) and Bonferroni/Dunnett's-T3 post hoc tests were used for statistical analyses (PASW Statistics 18.0). Significant differences were considered at p < 0.05 level.

## Results

### Toxicity of chemical inhibitors or potassium depletion in Sf 1 Ep cells

**Figure 3:**
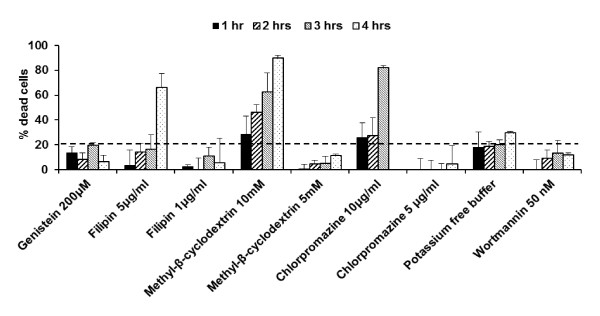
**Cellular toxicity of the endocytic inhibitors in Sf 1 Ep cells**. Cellular toxicity of chemical inhibitors/potassium depletion in Sf 1 Ep cells was measured at 1, 2, 3 and 4 hours. Conditions regarding concentration of the inhibitor and incubation time that retained cell viability greater than 80% were selected for cellular uptake study.

In order to optimize the concentration and the exposure time of the endocytosis inhibitors, we evaluated their intrinsic toxicity on Sf 1 Ep cells after 1, 2, 3 and 4 hours of incubation period (Figure 3). With the exception of 10 mM methyl-β-cyclodextrin and 10 μg/ml chlorpromazine concentrations, toxicity was under 20% at the 3-hour time point. Therefore, we selected a lower concentration of these inhibitors, 5 mM for methyl-β-cyclodextrin and 5 μg/ml for chlorpromazine which retained more than 80% cell viability up to 3 hours.

### Combined toxicity of P/G/L nanoparticles and chemical inhibitors/potassium depletion in Sf 1 Ep cells

**Figure 4:**
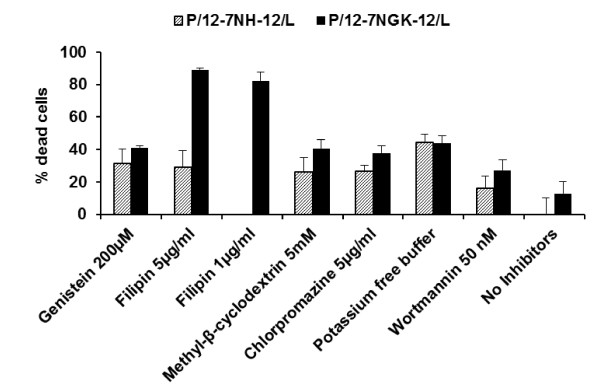
**Cellular toxicity of the endocytic inhibitors in the presence of P/G/L nanoparticles in Sf 1 Ep cells**. Combined cellular toxicity of P/12-7NH-12/L or P/12-7NGK-12/L in the presence of chemical inhibitors/potassium free buffer in Sf 1 Ep cells was measured at 72 hours. Filipin had a significantly high combined toxicity; therefore, it was not selected for gene expression study of the P/12-7NGK-12/L nanoparticles.

The inhibition of endocytosis by chemical treatments is a reversible process; therefore, it is important to maintain these inhibitors in cell media while transfecting the cells with the DNA nanoparticles [7,8,10,12]. Caveolae-mediated inhibitors (filipin and genistein) with P/12-7NH-12/L (parent gemini surfactant) exhibited an increase in cytotoxicity with 20-40% dead cells (Figure 4). In the presence of P/12-7NGK-12/L (amino acid-substituted gemini surfactant), filipin caused a 89.1 ± 0.8% cytotoxicity. A decrease of the filipin concentration to 1 μg/ml did not improve cell viability significantly (82.4 ± 5.2% dead cells), thus filipin could not be used as an endocytosis inhibitor due to the significant combined toxicity with the P/G/L nanoparticle. However, genistein, another inhibitor of the caveolae-mediated uptake had a cell toxicity of 41.2 ± 1.1% in the presence of P/12-7NGK-12/L, in the range of other agents. Methyl-β-cyclodextrin which inhibits both clathrin- and caveolae- dependent endocytosis also increased cytotoxicity with 26.3 ± 8.9% and 40.6 ± 5.4% cell death in presence of P/12-7NH-12/L and P/12-7NGK-12/L nanoparticles, respectively.

Clathrin-mediated uptake inhibition (potassium depletion, chlorpromazine) along with P/G/L nanoparticles showed an increase in toxicity to 20-45% cell death. In potassium depleted cells, the cytotoxicity values were 44.6 ± 4.8% of dead cells in the presence of P/12-7NH-12/L and 43.8 ± 4.7% dead cells after treatment with P/12-7NGK-12/L. Cytotoxicity was 26.6 ± 8.9% in cells incubated with chlorpromazine and P/12-7NH-12/L, while it was 37.9 ± 4.6% in the presence of P/12-7NGK-12/L. Wortmannin marginally increased cytotoxicity to 16.1 ± 7.6% and 27.2 ± 6.5% in the presence of P/12-7NH-12/L and P/12-7NGK-12/L, respectively. With the exception of filipin in the presence of P/12-7NGK-12/L, the cell toxicity among endocytosis inhibitors was not significantly different, thus their intrinsic toxicity was not considered as a modifying factor in gene expression. Due to the higher toxicity of filipin, genistein was selected to correlate gene expression to the caveolae-dependent pathway of the P/12-7NGK-12/L nanoparticles.

### Cellular uptake of P/G/L nanoparticles in cells treated with chemical inhibition/potassium depletion

**Figure 5:**
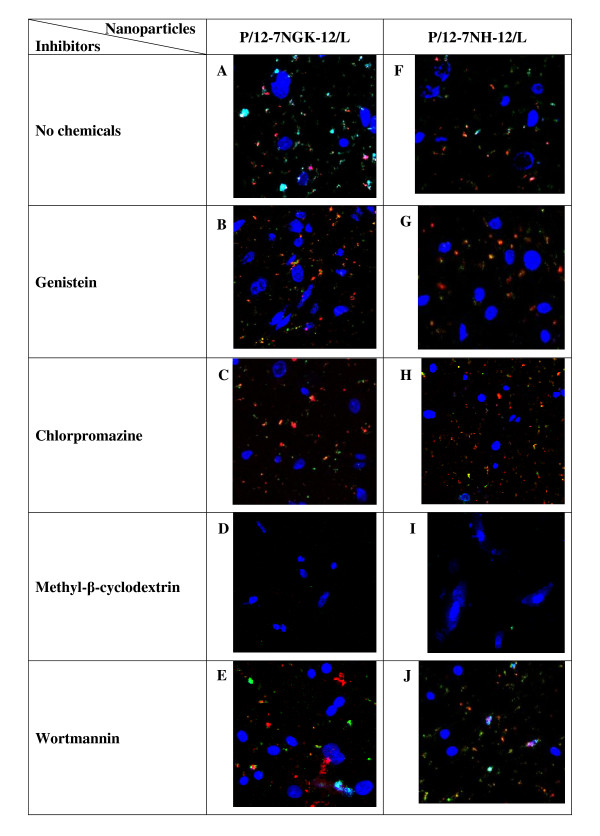
**Laser scanning confocal microscopy (LSCM) images of Sf 1 Ep cells**. Cells were pretreated with genistein (B & G), chlorpromazine (C & H), methyl-β-cyclodextrin (D & I) and wortmannin (E & J). Cells (A to E) were incubated with P/12-7NGK-12/L and Cells (F to J) were incubated with P/12-7NH-12/L for 2 hours. DAPI was used for staining the cell nuclei. At the end of the study, cells were washed with PBS and images were recorded. DNA (red), DOPE (green) and cell nuclei (blue).

P/G/L nanoparticles were prepared using TM-rhodamine (red) labeled plasmid in the presence of fluorescent (green) labeled helper lipid. DAPI (blue) was used as fluorescent stain for the cell nuclei. The objective was to determine, qualitatively, whether materials were transferred into the cells and whether the presence of inhibitors induced a difference in the cellular uptake of P/12-7NH-12/L and P/12-7NGK-12/L nanoparticles. Interestingly, we observed that the inhibition of caveolae-mediated (genistein) or clathrin-mediated (chlorpromazine) uptake pathway did not have any effect on cellular uptake of P/12-7NGK-12/L or P/12-7NH-12/L nanoparticles (Figure 5B &5G, 5C &5H). However, almost no particles were internalized by the cells treated with either P/12-7NGK-12/L or P/12-7NH-12/L nanoparticles when both clathrin-mediated and caveolae-mediated uptake pathways were inhibited by prior treatment with methyl-β-cyclodextrin (Figure 5D &5I). This suggests that both clathrin-mediated and caveolae-mediated pathways are playing an important role in uptake of these particles. Wortmannin (Figure 5E &5J) showed no difference in the cellular uptake of P/12-7NGK-12/L or P/12-7NH-12/L nanoparticles.

### Gene expression of P/G/L nanoparticles in cells treated with chemical inhibition/potassium depletion

In our previous studies, we found that the amino acid-substitution on the parent gemini surfactant increased its transfection efficiency [21]. Here, we evaluated, quantitatively, the effect of each uptake pathway on gene expression of P/12-7NH-12/L and P/12-7NGK-12/L. Nanoparticles internalized via caveolae-mediated, clathrin-mediated and macropinocytosis encounter different intracellular conditions; however, in each case the particles should escape degrading conditions such as lysosomes for successful gene expression. The gene expression levels achieved after inhibiting each cellular uptake pathway, separately, provides information on efficiency of nanoparticles in escaping these detrimental conditions.

#### Caveolae-mediated uptake

**Figure 6:**
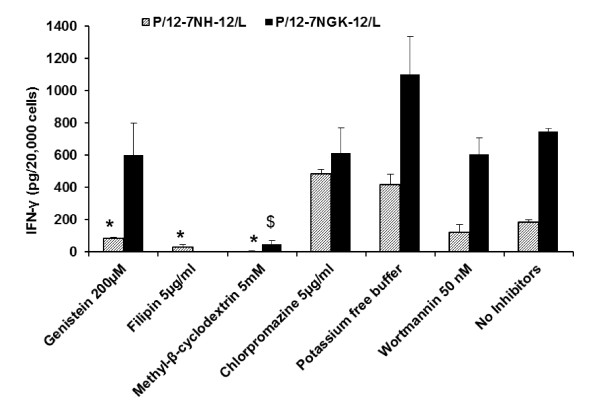
**Effect of endocytic inhibitors on gene expression of P/G/L nanoparticles**. Interferon (IFN)-γ expression was measured at 72 hours in cells incubated with P/G/L nanoparticles in the presence of endocytic inhibitors. In the P/12-7NH-12/L-treated cells, gene expression in cells pre-treated with caveolae-mediated uptake inhibitors (genistein, filipin) was significantly lower than the untreated (no inhibitors) cells, whereas gene expression was significantly higher on inhibiting clathrin-mediated uptake (chlorpromazine). For the P/12-7NGK-12/L nanoparticles, gene expression was significantly lower in presence of methyl-β-cyclodextrin (clathrin- and caveolae-mediated uptake inhibitor). * represents significant difference, p < 0.05, compared to 'No inhibitors' control cells treated with P/12-7NH-12/L nanoparticles (No inhibitors black bar). $ represents significant difference, p < 0.05, compared to 'No inhibitors' control cells treated with P/12-7NGK-12/L nanoparticles (No inhibitors dashed-line bar).

In the absence of any inhibitor treatment to the cells, the gene expression in the P/12-7NH-12/L transfected cells was 183 ± 17.5 pg IFNγ/20000 cells (Figure 6). Gene expression after P/12-7NH-12/L nanoparticle transfection was 84 ± 9.4 pg IFNγ/20000 cells and 31 ± 14.9 pg IFNγ/20000 cells in the presence of genistein and filipin, respectively, significantly lower (p < 0.05) compared to the cells treated with the P/G/L in the absence of the inhibitors.

The cells treated with the glycyl-lysine substituted nanoparticles (P/12-7NGK-12/L), exhibited gene expression of 749 ± 15.2 pg IFNγ/20000 cells in the absence of inhibitors. This gene expression was not significantly affected by inhibition of caveolae-mediated uptake (genistein), exhibiting protein levels of 601 ± 200 pg IFNγ/20000 cells. Unfortunately, due to high toxicity of filipin in the presence of P/12-7NGK-12/L (Figure 4), the gene expression data was not used for analysis. Methyl-β-cyclodextrin, that inhibits both clathrin- and caveolae-mediated uptake, reduced significantly (p < 0.05) gene expression in cells transfected with P/12-7NH-12/L and P/12-7NGK-12/L nanoparticles to 6 ± 2.2 pg IFNγ/20000 cells and 50 ± 21.1 pg IFNγ/20000 cells (Figure 6), respectively.

In conclusion, this suggests that caveolae-mediated uptake was crucial for successful gene expression of P/12-7NH-12/L nanoparticles. However, inhibition of caveolae-mediated uptake alone lowered slightly, but not significantly, the gene expression in the cells transfected with the P/12-7NGK-12/L nanoparticles.

#### Clathrin- mediated uptake

It was interesting to note that gene expression of P/12-7NH-12/L nanoparticles was significantly higher (p < 0.05) after clathrin-mediated uptake inhibition, compared to cells without inhibitors (Figure 6). The gene expression increased to 483 ± 30.6 pg IFNγ/20000 cells in the cells pre-treated with chlorpromazine. Potassium depletion, which also inhibits clathrin-mediated uptake, increased gene expression of P/12-7NH-12/L to 415 ± 70 pg IFNγ/20000 cells.

Inhibition of clathrin-mediated uptake by chlorpromazine slightly reduced gene expression of P/12-7NGK-12/L to 613 ± 159 pg IFNγ/20000 cells while gene expression was moderately increased to 1104 ± 235 pg IFNγ/20000 cells after potassium depletion. However, the effect of inhibition of clathrin-mediated uptake on gene expression of P/12-7NGK-12/L was not significant compared to cells without inhibitors. As stated above, inhibition of both clathrin-mediated and caveolae-mediated uptake with methyl-β-cyclodextrin reduced the gene expression of P/12-7NGK-12/L significantly (p < 0.05) compared to the control transfected cells.

In conclusion, P/12-7NH-12/L nanoparticles internalized via clathrin-mediated uptake were not a major contributor to overall gene expression. On the contrary, P/12-7NGK-12/L nanoparticles following either clathrin- or caveolae- mediated pathway resulted in successful gene expression.

#### Macropinocytosis

Macropinocytosis inhibitor, wortmannin, did not significantly modify gene expression of either P/12-7NH-12/L or P/12-7NGK-12/L (Figure 6). Protein levels of 121 ± 49 pg IFNγ/20000 cells and 606 ± 104 pg IFNγ/20000 cells were measured for P/12-7NH-12/L and P/12-7NGK-12/L, respectively.

### Particle size measurement of P/G/L nanoparticles as a function of pH

**Figure 7:**
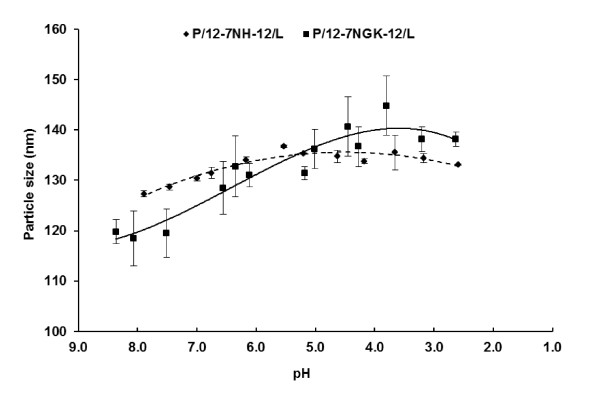
**Particle size measurement of P/12-NH-12/L and P/12-NGK-12/L at different pH**. A second order polynomial curve (solid line) was found to be a best fit (R^2 ^> 0.9) for P/12-NH-12/L while a third order polynomial (dashed line) curve (R^2 ^> 0.9) described the effect of pH on P/12-NGK-12/L particle size. Stronger acidic conditions resulted in a significant increase in P/12-NGK-12/L particle size.

The pathway of cellular uptake determines the intracellular fate of the engulfed particle [4]. Cellular internalization of the particles via clathrin-coated endosomes proceeds with an early influx of H^+ ^before fusion with the late endosome, finally merging with the lysosomes. In order to simulate these conditions, we evaluated the behavior of the P/G/L complexes over the pH range of 8.0-3.0. Particle size increased in the pH range 8.0-5.5 followed by a decreasing trend (Figure 7), fitting a second order polynomial (quadratic) relationship (R^2 ^> 0.9) between pH and particle size of P/12-NH-12/L. The P/12-7NGK-12/L nanoparticles followed a third order polynomial (cubic) curve exhibiting an exponential increase in particle size from neutral (~7.4) to acidic (~4) pH. The increase in particle size of P/12-7NGK-12/L nanoparticles could be attributed to the accumulation of H^+ ^ions due to the higher buffering capacity of the amino acid-substituted gemini surfactant.

**Figure 8:**
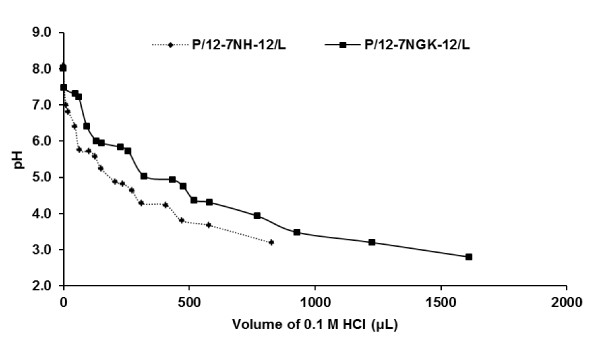
**Comparison of buffering capacity of P/G/L nanoparticles**. **A **pH titration curve of P/G/L nanoparticles exhibited considerably higher buffering capability of P/12-7NGK-12/L compared to P/12-7NH-12/L in neutral, slightly acidic and strong acidic pH.

The volume of acid (0.1 M HCl) used per unit pH value provided a relative comparison of the buffering ability of 12-7NGK-12 and 12-7NH-12. The pH titration curve revealed that P/12-7NGK-12/L nanoparticles had considerably higher buffering capacity than P/12-7NH-12/L at neutral (~7.4), slightly acidic (6-5) and strongly acidic (~4) pH values (Figure 8).

### Interactions between the DNA and gemini surfactants

Ethidium bromide exclusion assay is a useful technique to study DNA/delivery vector interaction. The assay was performed in the presence of the polyanionic heparin, which competes with DNA to bind with the cationic gemini surfactants. The interaction between the gemini surfactants and heparin could cause a dissociation of the P/G/L complexes, allowing the ethidium bromide to intercalate in the DNA strand and emit fluorescence. Lower fluorescence values indicate stronger binding property of DNA/gemini surfactants and a higher protection of the genetic material. Also, stronger binding properties ensure that DNA is not released prematurely, upon interaction with negatively charged cell surface proteins.

**Figure 9:**
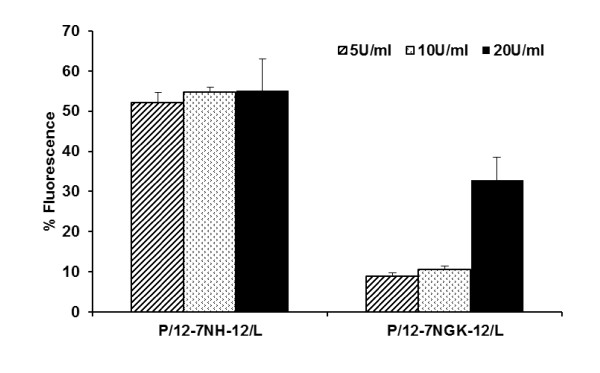
**DNA-gemini surfactant binding properties**. An ethidium bromide dye exclusion assay was performed to determine the binding efficiency of 12-7NH-12 and 12-7NGK-12 with plasmid DNA in the presence of DOPE and heparin (5 U/ml, 10 U/ml and 20 U/ml). At lower concentration of heparin (polyanions), amino-acid substituted gemini exhibited stronger DNA binding compared to unsusbtituted gemini surfactant. At higher concentration (20 U/ml heparin), a significant increase in fluorescence was observed, indicating dissociation of DNA and 12-7NGK-12.

At the 1:10 charge ratio of DNA and gemini surfactant in the presence of DOPE, a small increase in fluorescence (8.9 ± 0.8%) was observed in P/12-7NGK-12/L nanoparticles upon addition of polyanions (5 U/ml heparin), exhibiting a stronger protection of DNA compared to the parent gemini surfactant which had an increase of 52.3 ± 2.3% fluorescence (Figure 9). A similar trend occurred at higher concentrations of 10 U/ml and 20 U/ml of heparin. Interestingly, a steady increase in fluorescence was observed in P/12-7NGK-12/L nanoparticles with a significant spike in fluorescence (32.9 ± 5.7%) occurring in the presence of 20 U/ml heparin. This indicates that 12-7NGK-12 has balanced DNA binding properties which ensures protection of DNA as well as an ability to interact with a highly negative charged membrane, such as the inside of caveolae endosomes. These balanced binding properties (protection and release) may be instrumental in efficient endosomal escape leading to higher transfection efficiency of P/12-7NGK-12/L compared to the P/12-7NH-12/L nanoparticles (Figure 6).

### Transmission electron microscopy (TEM) imaging

**Figure 10:**
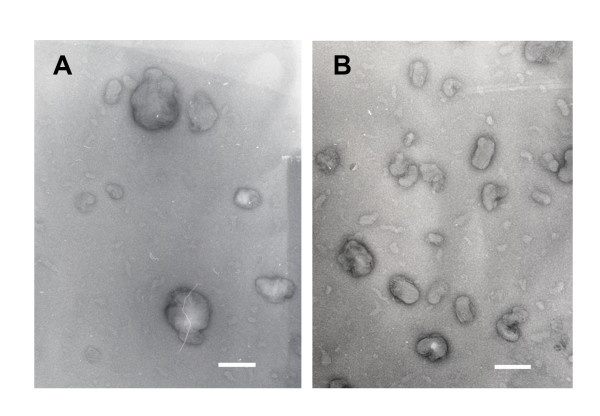
**Transmission electron microscopy (TEM) images of P/G/L complexes**. (A) P/12-7NH-12/L nanoparticles (B) P/12-7NGK-12/L nanoparticles. P/12-7NGK-12/L nanoparticles are cylindrical while P/12-7NH-12/L are spherical in shape. Scale bar corresponds to 200 nm.

Particle shape and particle aggregation behavior can also influence the gene expression of nanoparticles. Micrographs of the P/12-7NH-12/L and P/12-7NGK-12/L nanoparticles showed consistency with the previous light scattering measurement, with particles of 100-150 nm in diameter being observed. The TEM images showed no aggregation behavior of the particles (Figure 10). However, the P/12-7NGK-12/L nanoparticles were found to be more cylindrical shaped, while the P/12-7NH-12/L particles showed spherical morphology.

## Discussion

In non-viral gene delivery, cellular uptake of the DNA nanoparticles can influence the overall gene expression due to the number of particles that are internalized and the intracellular trafficking of the engulfed particle. In our previous work, we found that glycyl-lysine substitution in the spacer region of the gemini surfactants improved gene expression, and the transfection efficiency correlated with DNA binding properties of gemini surfactants [21]. In this study, we evaluated whether the pathway of the cellular uptake of the P/12-7NH-12/L and P/12-7NGK-12/L nanoparticles in Sf 1 Ep cells is a major factor affecting the transfection efficiency of these novel delivery systems. Clathrin-mediated uptake, caveolae-mediated uptake and macropinocytosis are known to be three of the most important uptake pathways in mammalian cells [4]. Treatment with chemical inhibitors such as genistein, filipin, methyl-β-cyclodextrin, chlorpromazine, wortmannin, and potassium free buffer are routinely used to determine the effect of each pathway on cellular uptake of nanoparticles [8,12,22-24]. Upon treatment of the Sf 1 Ep cells with these chemicals at different concentrations, we selected those agents that showed high cell viability with no significant difference among the inhibitors, thus minimizing the impact of the agents on the overall gene expression. Conditions regarding concentration of the inhibitor and incubation time that retained cell viability greater than 80% were selected for further experiments (Figure 3). In previous studies, we found that the P/G/L nanoparticles were safe, inducing minimal toxicity (9-12%) to the cells [21]; however, the combined toxicity of P/G/L nanoparticles and chemical inhibitors/potassium depletion was unknown. The cell toxicity induced by the chemical treatment in the presence of P/G/L nanoparticles increased to 30-40% (Figure 4). Without a significant difference in the toxicity among various agents, the cellular uptake study in Sf 1 Ep cells was found to provide meaningful insight of the mechanism of uptake and shed light on the crucial relationship between the physicochemical parameters and the difference in the transfection efficiency of the parent and glycyl-lysine substituted gemini nanoparticles.

In the cellular uptake study using CLSM, it was observed that the inhibition of caveolae-mediated (genistein) or clathrin-mediated (chlorpromazine) uptake did not influence significantly the uptake of P/12-7NGK-12/L or P/12-7NH-12/L nanoparticles. However, no particles were internalized upon inhibition of both clathrin-mediated and caveolae-mediated pathways, simultaneously, by methyl-β-cyclodextrin (Figure 5D &5I). This suggests that P/G/L nanoparticles were able to recruit both clathrin-mediated and caveolae-mediated uptake. In previous studies, cationic lipid-DNA complexes (using DOTAP and SAINT-2/DOPE) have been suggested to preferentially internalize via clathrin-mediated endocytosis [22,25]. However, more recent studies emphasized that the physiochemical parameters of the DNA complexes (such as particle size, shape and surface chemistry) as well as cell type can influence the uptake behavior [26-28]. Polyethylenimine (PEI)/DNA complexes were found to internalize via clathrin- or caveolae-mediated mechanisms depending on both PEI type (linear or branched) and cell line [28]. Interestingly, another study using cationic lipid, Amphiphile 1 and DNA complexes found macropinocytosis to be the major pathway leading to gene transfection in CHO cells [29]. Caveolae-mediated uptake was also found to be important for transfection of human serum albumin coated DOTAP/DOPE complexes [30]. In the present study, the role of caveolae-mediated uptake in cationic gemini surfactant based DNA nanoparticles was confirmed by a drop in gene expression in cells transfected with P/12-7NH-12/L in the presence of genistein and filipin, 54% (genistein) and 83% (filipin), compared to the control. Inhibition of clathrin-mediated uptake (chlorpromazine, potassium depletion) did not reduce the gene expression of P/12-7NH-12/L; on the contrary, it led to an increase (more than double) in gene expression. There may be two reasons for this: (a) inhibition of the clathrin-mediated uptake results in an up-regulation of other uptake pathways as observed in certain mammalian cell types [31] or (b) inhibition of the clathrin-mediated pathway leads to the availability of more nanoparticles to follow the caveolae-mediated uptake, or a combination of both. P/12-7NH-12/L nanoparticles following the intracellular route via clathrin-mediated uptake may not be contributing substantially in overall gene expression. No gene expression was observed upon inhibition of both clathrin-mediated and caveolae-mediated pathway by methyl-β-cyclodextrin. Macropinocytosis (wortmannin) had no significant effect on the gene expression in cells transfected with the P/12-7NH-12/L nanoparticles. While there was only a modest decrease in the gene expression in the P/12-7NGK-12/L transfected cells after inhibition of clathrin- or caveolae-mediated uptake, gene expression decreased by 93% when both pathways were blocked simultaneously by methyl-β-cyclodextrin. This suggests that P/12-7NGK-12/L nanoparticles can be internalized via either clathrin-mediated or caveolae-mediated routes, are able to avoid degradation, and release DNA at appropriate time for successful gene expression.

Analysis of gene expressions also pointed to an interesting fact: glycyl-lysine substitution not only improved the overall transfection efficiency of P/12-7NGK-12/L but also the gene expression level via each route (clathrin-mediated or caveolae-mediated) was higher than P/12-7NH-12/L. This difference in gene expression may be due to the number of particles that are internalized or improved endosomal escape of P/12-7NGK-12/L particles. Particle size and shape may affect cellular uptake, significantly [27]. As reported previously, the particle size of the P/12-NGK-12/L and P/12-NH-12/L nanoparticles was 117.3 ± 0.6 nm and 133.0 ± 2.8 nm, respectively [20]. The smaller particle size of P/12-7NGK-12/L might be advantageous in improving the rate of cellular uptake. Rod-like or cylinder shaped particles were found to be internalized faster than the spherical particles [27]. The effect of cylindrical morphology is more significant on the cellular uptake when the aspect ratio (diameter to width) is high, especially in particles with diameter > 150 nm [27]. TEM images showed that P/12-7NH-12/L particles were cylindrical while P/12-7NGK-12/L particles were spherical (Figure 10). A large number of particles ensure availability of more DNA particles inside the cell. However, this does not guarantee higher gene expression since the DNA can be degraded in the cytosol. Therefore, we further analyzed the important physicochemical properties that may be contributing to more efficient intracellular trafficking of these nanoparticles.

### Glycyl-lysine substitution improves endosomal escape in clathrin-mediated pathway

Intracellular trafficking of an engulfed particle is influenced by the type of uptake. In clathrin-mediated endocytosis, the nanoparticles are entrapped in endosomes, transported to late endosomes, and finally merged with lysosomes for complete degradation. This latter step, degradation of the genetic material in the lysosomes, can significantly decrease the overall availability of plasmid DNA for translocation to the nucleus for gene expression [32]. However, several scenarios can be envisioned to overcome this challenge by utilization of an interesting phenomenon that happens during the transfer of cargo from early endosomes to late endosomes. In mammalian cells, there is an early drop in pH in the clathrin-coated endosomes as they travel towards the late endosomes, creating strong acid conditions before merging with the lysosomes [6]. These conditions can be used in an advantageous manner by designing pH-sensitive delivery vectors that can either absorb H^+^-ions or unfold (relax) in an acidic environment. In the former case, a significant increase in H^+^-ions will trigger an increased inflow of aqueous medium leading to swelling and then bursting of endosomes, releasing the DNA nanoparticles into the cytoplasm [33]. In the latter case, the nanoparticles can unfold and increase their size, significantly, leading to a mechanical disruption of the endosomal membrane and rescuing the DNA from lysosomal degradation [34].

**Figure 11:**
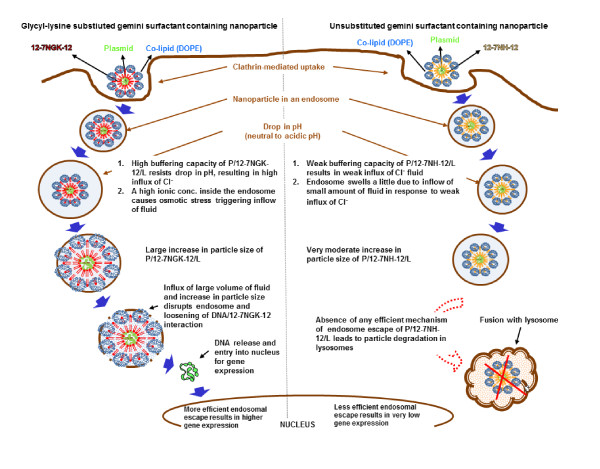
**Intracellular trafficking of P/G/L complexes via clathrin-mediated pathway**. A schematic comparison of crucial steps in endosomal escape of P/12-7NGK-12/L (on the left side of the image) and P/12-7NH-12/L (on the right side of the image) nanoparticles in clathrin-mediated cellular uptake.

In the clathrin-mediated uptake, we found that buffering capacity (Figure 8) and particle size increment (Figure 7) over neutral to acidic pH may be two important properties that could be causing P/12-7NGK-12/L nanoparticles to escape more efficiently than the P/12-7NH-12/L, and lead to timely release of plasmid for nuclear localization and gene expression. In the slow but steady decrease of pH, the P/12-7NGK-12/L nanoparticles could absorb more protons triggering an inflow of Cl^-^. The flow of ions also brings more fluid inside the endosomal vesicle, ultimately leading to its breakdown and release of the DNA into the cytoplasm. This scenario is supported by the fact that, at physiological pH, the size of the P/12-7NGK-12/L nanoparticles was 119.5 ± 4.8 nm which then increased significantly with a drop in pH (Figure 7). Maximum particle size of 144.8 ± 5.9 nm was found at strongly acidic conditions (~pH 4), a net increase of 21.2% compared to the original particle size. On the other hand, the parent compound had a particle size of 128.6 ± 0.6 nm at physiological pH with a maximum size of 136.7 ± 0.3 nm around 5-5.5 pH (Figure 8). This is an overall increase of only 6.3% compared to the original particle size which may not have been instrumental in endosomal escape. The decrease in pH in the endosomes could allow for additional interaction with the endosomal membrane and promote DNA escape/release (Figure 11). This increase in particle size could be an indication of the relaxation of the nanoparticles; facilitating the availability of more 12-7NGK-12 molecules for absorption of H^+^, thus, increasing overall buffering capacity of the system. The increase in particle size may also be an indication of reduced interaction between the DNA and 12-7NGK-12, facilitating DNA release from the complexes after endosomal escape, as suggested by increased ethidiun bromide intercalation in the presence of high concentrations of polyanion (Figure 9).

### Glycyl-lysine substitution improves DNA/gemini surfactant binding properties

Previously, we found that DNA/gemini surfactant interactions play a crucial role in gene delivery ability of these molecules [21]. Conformational flexibility achieved by insertion of a glycine moiety as a linking molecule between the spacer region and terminal lysine lead to a balance between DNA binding and release behavior of the P/12-7NGK-12/L nanoparticles. In this study, we further explored this idea in the context of intracellular trafficking following caveolae-mediated uptake of the P/G/L nanoparticles.

**Figure 12:**
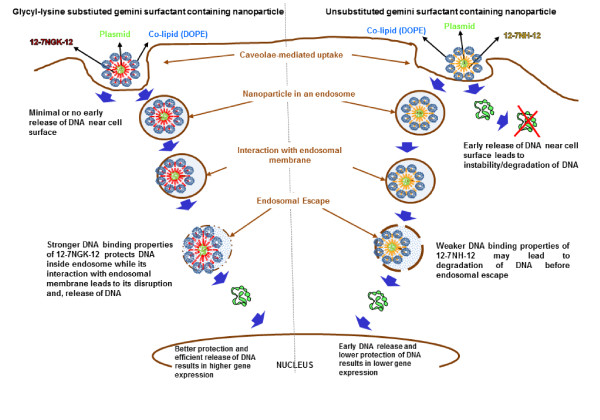
**Intracellular trafficking of P/G/L complexes via caveolae-mediated pathway**. A schematic comparison of crucial steps in endosomal escape of P/12-7NGK-12/L (on the left side of the image) and P/12-7NH-12/L (on the right side of the image) nanoparticles in caveolae-mediated cellular uptake.

In caveolae-mediated uptake, there is no gradual pH transition that could influence the interaction between the gemini surfactants and DNA. Therefore, we mimicked this pathway by evaluating the behavior of the nanoparticles in the presence of a polyanion (heparin) which could potentially compete for the cationic charges on the gemini surfactants with the DNA, and determined its effect on DNA binding properties of the gemini surfactants (Figure 12). We found that low concentrations of heparin with P/12-7NGK-12/L did not affect binding of the gemini surfactant to DNA, significantly, compared to nanoparticles formulated with the parent gemini surfactant. The shielding is crucial for the initial interaction of P/G/L nanoparticles with the cell surface membrane and uptake without premature release of the DNA at the cell surface. However, at higher concentration of heparin, the accessibility of the DNA in P/12-7NGK-12/L increased significantly (Figure 9). This suggests that the 12-7NGK-12/L lipid system provides balanced DNA binding properties that enable the 12-7NGK-12/L system to protect the DNA as well as interact readily with the anionic proteins coating the inner surface of the caveolae-mediated vesicle (endosome). This balance may be instrumental in the efficient disruption of vesicle membrane leading to DNA escape.

## Conclusion

Amino-acid substitution in the spacer of the gemini surfactant did not alter the cellular uptake pathway, showing a similar pattern to the unsubstituted parent gemini surfactant. Both clathrin-mediated and caveolae-mediated pathways were equally important routes of uptake for P/G/L nanoparticles. Glycyl-lysine substitution in the gemini spacer improved buffering capacity and imparted a pH-dependent increase of particle size. This property conferred to the P/12-7NGK-12/L nanoparticles the ability to escape efficiently from clathrin-mediated endosomes and leads to higher transfection efficiency compared to the parent gemini surfactant. More balanced binding properties (protection and release) of P/12-7NGK-12/L in the presence of polyanions play an important role in the release of nanoparticles internalized via caveolae-mediated uptake. These factors (balanced binding properties and improved intracellular endosomal escape) will be taken into consideration in the design of further structural modifications, such as attachment of other basic amino acids via glycyl linkage to the gemini surfactants of the P/G/L nanoparticles.

## Competing interests

The authors declare that they have no competing interests.

## Authors' contributions

Conceived and designed the experiments: JS, IB. Performed the experiments: JS, DM. Analyzed the data: JS. Contributed materials: JMC, REV. Wrote the paper: JS, REV, IB. All authors read and approved the final manuscript

## Funding

This work was supported by Saskatchewan Health Research Foundation (SHRF), Canada; Natural Sciences and Engineering Research Council (NSERC), Canada; and the Drug Design and Discovery Research Group, College of Pharmacy and Nutrition, University of Saskatchewan, Canada.
